# Word complexity modulates the divided-word effect during Chinese reading

**DOI:** 10.3389/fpsyg.2022.921056

**Published:** 2022-09-23

**Authors:** Mingzhe Zhang, Xuejun Bai, Sainan Li

**Affiliations:** ^1^Faculty of Psychology, Tianjin Normal University, Tianjin, China; ^2^Academy of Psychology and Behavior, Tianjin Normal University, Tianjin, China

**Keywords:** divided-word effect, word complexity, stroke number, eye movements, Chinese reading

## Abstract

The present study examined the influence of word complexity on the divided-word effect. By manipulating presentation conditions (line-final presentation vs. divided-word presentation vs. line-initial presentation) and visual complexity (high vs. low), we found a significant divided-word effect that the reading times such as gaze duration and total reading time were significantly longer in the divided-word presentation condition than in both the line-final and line-initial presentation conditions. On the measure of total reading time, the marginally significant interaction between the divided-word versus line-final presentation comparison and complexity showed that the divided-word effect was larger for low complexity words than that for high complexity words. These results suggest that dividing a word across two lines interferes with reading, and word complexity modulates this effect.

## Introduction

In Chinese, most words are composed of one to four characters, however, there is no obvious word boundary during Chinese text or paragraph reading (Reichle and Yu, 2018). Those multi-character words are often divided across two lines in paragraphs. For example, according to the survey of the essays of a zoology class of 30 students, among the 768 lines that contained a multiple-character word (or part of a multiple-character word) at the line end, 37% of the words are divided across two lines (Li et al., 2012).

Does dividing words across two lines influence readers’ reading performance? There may be two cases for this question. The first is that dividing words across two lines does not interfere with reading behavior. Unlike English, there is no hyphen to connect words in Chinese when a word is split across lines. The reason may be that there are no spaces between words in Chinese. The lack of clear word boundaries, combined with the fact that characters themselves are orthographically distinct and spatially discrete, leads to the claim that Chinese readers adopt a character-based reading strategy (Yu et al., 2021). If Chinese readers process characters one by one, then dividing a word across two lines should not influence Chinese reading.

However, this may also be the case that dividing a word possibly interferes with the holistic processing of words, thereby slowing down word recognition. Many studies have shown that words are important in Chinese reading and Chinese readers adopt a word-based reading strategy (Zang et al., 2018). The evidence comes from the following studies: first, word properties affect eye movements such that low-frequency and unpredictable words elicit longer fixation durations and outgoing saccades (Wei et al., 2013; Liu et al., 2017, 2018; Chang et al., 2020). Second, using word space or color to mark word boundaries is beneficial to reading (Liu and Lu, 2018; Zhou et al., 2020; Pan et al., 2021). Finally, the word-superiority effect showed that character recognition is faster in real words than in nonwords (Chen et al., 2018). These studies suggest that words act as the basic processing units in Chinese reading (Zhou and Li, 2021; Li et al., 2022). When readers cannot simultaneously process all the characters belonging to a word, reading performance will be disturbed (Li et al., 2013). Based on the studies reviewed above, it can be anticipated that dividing a word across lines should interfere with reading.

Li et al. (2012) found that dividing a word across two lines interferes with Chinese reading. In their study, they manipulated the character position of the last words in experimental lines: the divided-word presentation condition in which the first character is located at the line-final position, but the second character is located at the line-beginning position, and the line-final presentation condition in which both characters belonging to a two-character word is presented in the line-final position. They found that fixation durations were longer in the divided-word presentation condition than in the line-final presentation condition, namely the divided-word effect, which indicated that dividing words into two lines disturbs reading. When examining the divided-word effect, there are usually three presentation conditions: besides the divided-word presentation and line-final presentation condition, the line-initial presentation condition is also needed, in which both characters belonging to a two-character word are presented in the line-initial position. To convincingly demonstrate the divided-word effect, we also need further evidence that fixation durations are longer in the divided-word condition than in the line-initial presentation condition. The reason is that readers not only acquire information from the currently fixed words but also from the right of the fixed word, namely parafoveal vision, which is beneficial for proficient reading (Rayner et al., 2016; Vasilev and Angele, 2017; Andrews and Veldre, 2019). When a word is presented at the line-final position, readers can acquire a complete preview of this word, but when a word is divided across lines, readers only obtain preview information of the initial part of this word, because its final part is at the beginning of next line in the peripheral vision. Less preview benefit may lead to longer fixation duration in the divided-word presentation condition than in the line-final presentation condition. However, when a word is presented at the line-beginning position, which is located in the peripheral vision, readers cannot process this word in parafovea. If fixation duration is also longer in the divided-word presentation condition than in the line-initial presentation condition, this result may convincingly demonstrate that dividing a word across lines interferes with reading.

To understand the universality of the divided-word effect, Li et al., also controlled the difficulty level of the passages: easy, medium, and difficult. They found no interaction between presentation condition and text difficulty level, suggesting that dividing a word across two lines only affects the very low-level word recognition process. However, it is worth noting that they only manipulated the overall text difficulty level in which target words could be easy or difficult in different difficult text levels. So, it is not clear whether word difficulty (e.g., visual complexity) influences the divided-word effect if only the target word difficulty level is manipulated. The choice of visual complexity as an index of word difficulty is mainly because it is a low-level visual property (Zhang et al., 2020). The visual complexity of target words is generally measured in terms of the number of strokes (Su and Samuels, 2010; Yan, 2015). Many studies have found that fixation durations were longer, and the skipping rate was lower for high-than for low-complexity words (Liversedge et al., 2014; Ma and Li, 2015; Zang et al., 2016; Li et al., 2019). Visual simple words are easier to process as a whole, and dividing a word across lines disturbs the holistic processing of words. Therefore, it can be expected that dividing a word across lines has a greater impact on visual simple words, namely a larger divided-word effect for visual simple words.

In summary, the present study manipulates presentation conditions (line-final presentation vs. divided-word presentation vs. line-initial presentation) and visual complexity (high vs. low) to explore whether the divided-word effect exists in Chinese and how visual complexity modulates the divided-word effect. If fixation durations are longer in the divided-word presentation condition than in both the line-final and line-initial presentation condition, this can strongly demonstrate that dividing a word across lines interferes with reading in Chinese. But, if fixation durations for the divided-word presentation condition are longer than for the line-final presentation condition, but is shorter than the line-initial presentation condition, this may be only caused by the parafoveal preview difference. Since visual simple words are more inclined to process as a whole, it is anticipated that the divided-word effect is stronger for low-complexity than for high-complexity words.

## Materials and methods

### Participants

Forty-eight native Chinese-speaking students (6 males; mean age 20.3 years, age range 18–25 years) from Tianjin Normal University were paid to participate in the experiment. All participants had either normal or corrected-to-normal vision. Informed consent was obtained from the participants, and the study was approved by the Research Ethics Board of our university.

### Stimuli and design

One twenty pairs of high-and low-complexity two-character words were chosen as target words. Characters in high-complexity target words had at least nine strokes, while characters in low-complexity target words had seven or fewer strokes. Each pair of target words was embedded into the same sentence frame. Twenty participants who did not take part in the experiment assessed the naturalness of sentences on a five-point scale (1 = very unnatural, 5 = very natural). To determine the predictability of the target word, another 10 participants were asked to guess the next word using the sentence truncated immediately before the target word. The differences between high-complexity and low-complexity conditions were significant in word complexity, first character complexity, and second character complexity. Word frequency, first character frequency, second character frequency, predictability, and sentence naturalness were matched across different conditions (Table 1).

**Table 1:** Stimulus properties used in the experiment.

	Complexity		*t*	*p*
	High	Low		
*Target words*				
Word complexity (number of strokes)	24.98 (3.18)	10.59 (2.10)	39.00	<0.001
First character complexity (number of strokes)	12.42 (1.71)	5.28 (1.24)	35.90	<0.001
Second character complexity (number of strokes)	12.57 (2.35)	5.31 (1.51)	26.53	<0.001
Word frequency (counts per million)	23.53 (49.69)	22.93 (52.87)	0.17	0.87
First character frequency (counts per million)	464.93 (977.17)	633.63 (1035.95)	−1.46	0.15
Second character frequency (counts per million)	454.86 (1044.19)	627.49 (742.39)	−1.42	0.16
*Sentences*				
Predictability	0.009 (0.06)	0.003 (0.02)	1.07	0.29
Naturalness	4.30 (0.40)	4.30 (0.40)	0.11	0.92

The experiment sentences were displayed across two lines in 24-point Song font with double line spacing. There were 20 characters on average (ranges from 16 to 25 characters) before the target word appeared. Target words were presented in three different conditions. In the line-final presentation condition, both characters belonging to the target word were positioned at the end of the first line. In the divided-word presentation condition, the first character was positioned at the end of the first line, but the second character was at the start of the second line. In the line-initial presentation condition, both characters were positioned at the start of the second line.

The experiment was 3 (presentation condition: line-final presentation vs. divided-word presentation vs. line-initial presentation) × 2 (complexity: high vs. low) within-participant design. Six sentence sets were created, each containing 120 experiment sentences (20 sentences in each condition). According to Latin square, conditions were rotated across sentences so that participants could read each sentence frame only once. One-third of the sentences followed a “Yes/No” question. Table 2 shows an example sentence.

**Table 2:** Example sentence used in the experiment.

Condition	Sentence
Line-final – High complexity	小雪的父亲是位高级工程师，她多年来一直疑惑 父亲受过那么多的教育却那么专横。
Divided-word – High complexity	小雪的父亲是位高级工程师，她多年来一直疑 惑父亲受过那么多的教育却那么专横。
Line-initial – High complexity	小雪的父亲是位高级工程师，她多年来一直 疑惑父亲受过那么多的教育却那么专横。
Line-final – Low complexity	小雪的父亲是位高级工程师，她多年来一直纳闷 父亲受过那么多的教育却那么专横。
Divided-word – Low complexity	小雪的父亲是位高级工程师，她多年来一直纳 闷父亲受过那么多的教育却那么专横。
Line-initial – Low complexity	小雪的父亲是位高级工程师，她多年来一直 纳闷父亲受过那么多的教育却那么专横。

The sentence translates as “Xiaoxue has wondered for many years that her father, a senior engineer, is well-educated but so bossy.” The target words were shown underlined but displayed normally in the experiment. A yes-or-no question related to this sentence was: is Xiaoxue’s father reasonable?

### Apparatus

An SR Research EyeLink 1000 plus eye-tracking system with a sampling rate of 1,000 Hz was used to record participants’ eye movements during reading sentences. The sentences were presented on a 21-in CRT monitor (resolution, 1024 × 768 pixels; frame rate, 60 Hz). The distance between the monitor and the participants was 75 cm. At this distance, one character subtended approximately 0.9° per visual angle.

### Procedure

The experiment was conducted in a quiet room. First, participants were instructed to read the sentences silently for comprehension. Second, calibration and validation with a nine-point grid were performed until the error is < 0.5° of the visual angle. Third, a fixation point appeared at the location of the first character of the sentence for the drift check. Finally, sentences were presented. After finishing reading, participants need to press the Space key to initiate the next sentence or ask a question about this sentence. Before experiment sentences appeared, there were eight practice sentences.

### Data analysis

According to Li et al. (2012), the target word and the word before and after it were defined as a region of interest. The following eye-movement measures were used: (a) gaze duration (sum of all first-pass fixation durations); (b) total reading time (sum of all fixations); (c) total number of fixations (sum of the number of fixations).

Fixation durations that were longer than 1,200 ms or shorter than 80 ms were removed from the analysis. Trials with tracker loss or less than five fixations were also removed (0.8% of the data). Finally, we deleted observations for each measure that are above or below three standard deviations (0.7% of the data on average).

The eye movement measures were analyzed with linear mixed models using the “lmer” function of lme4 package (Bates et al., 2015) in R version 4.1.0 (R Core Team, 2021). Presentation condition and complexity were treated as fixed factors. For the presentation condition, sliding contrasts using the function “contr.sdif” in the MASS package (Venables and Ripley, 2002) were carried out with comparisons of line-final presentation vs. divided-word presentation, and divide-word presentation vs. line-initial presentation. Participants and items were treated as crossed random factors.

## Results

The mean accuracy of questions was 97%, which indicated that participants could comprehend the sentence well. A two-way repeated-measures analysis of variance (ANOVA) found that the main effects of presentation condition and complexity, as well as their interaction, were insignificant (all *p* > 0.6). The means and standard deviations of the eye-movement measures are displayed in Table 3.

**Table 3:** Means and standard deviations of the eye-movement measures (*M* ± *SD*).

	Gaze duration	Total reading time	Total number of fixations
*Line-final presentation*			
High complexity	633 ± 469	902 ± 596	3.82 ± 2.19
Low complexity	594 ± 437	850 ± 551	3.62 ± 2.05
*Divided-word presentation*			
High complexity	746 ± 515	996 ± 607	4.29 ± 2.27
Low complexity	711 ± 508	988 ± 610	4.21 ± 2.16
*Line-initial presentation*			
High complexity	645 ± 413	862 ± 508	3.69 ± 1.90
Low complexity	628 ± 416	842 ± 531	3.58 ± 1.99

### Gaze duration

Gaze durations were longer in the divided-word presentation condition than in the line-final presentation condition (*b* = 114.68, SE = 12.08, *t* = 9.50, *p* < 0.001) and than in the line-initial presentation condition (*b* = −92.74, SE = 19.78, *t* = −4.69, *p* < 0.001). High-complexity words received longer gaze duration than low-complexity words (*b* = −33.42, SE = 9.88, *t* = −3.38, *p* < 0.001).

There were neither significant interactions of the divided-word versus line-final presentation comparison with complexity (*b* = 4.34, SE = 24.15, *t* = 0.18, *p* = 0.857) nor significant interactions in the line-initial versus divided-word presentation comparison with complexity (*b* = 14.89, SE = 24.19, *t* = 0.62, *p* = 0.538).

### Total reading time

Total reading time were longer in the divided-word than in the line-final presentation condition (*b* = 120.53, SE = 13.36, *t* = 9.02, *p* < 0.001) and than in the line-initial presentation condition (*b* = −141.27, SE = 13.35, *t* = −10.58, *p* < 0.001). High-complexity words received longer total reading time than low-complexity words (*b* = −27.25, SE = 10.91, *t* = −2.50, *p* < 0.05).

The interaction between the divided-word versus line-final presentation comparison and complexity was marginally significant (*b* = 45.74, SE = 26.70, *t* = 1.71, *p* = 0.087). The difference between the divided-word presentation condition and the line-final presentation condition was larger for low-complexity words (*b* = 143.52, SE = 19.03, *t* = 7.54, *p* < 0.001) than for high-complexity words (*b* = 97.68, SE = 18.98, *t* = 5.15, *p* < 0.001), although the difference was significant for both low-and high-complexity word. There was no hint of a significant interaction between the line-initial versus divided-word presentation comparison and complexity (*b* = −10.91, SE = 28.09, *t* = −0.39, *p* = 0.698).

### Total number of fixations

The total number of fixations were greater in the divided-word than in the line-final presentation condition (*b* = 0.54, SE = 0.05, *t* = 10.33, *p* < 0.001) and in the line-initial presentation condition (*b* = −0.62, SE = 0.05, *t* = −11.75, *p* < 0.001). The total number of fixations on the high-complexity words were greater than low-complexity words (*b* = −0.13, SE = 0.06, *t* = −2.35, *p* = 0.021).

There were no interactions of the divided-word versus line-final presentation comparison with complexity (*b* = 0.12, SE = 0.11, *t* = 1.11, *p* = 0.269) and the line-initial versus divided-word presentation comparison with complexity (*b* = −0.03, SE = 0.10, *t* = −0.31, *p* = 0.758).

## Discussion

The present study manipulated the presentation condition (line-final presentation vs. divided-word presentation vs. line-initial presentation) and complexity (high vs. low) to explore the divided-word effect and the influence of complexity on the divided-word effect. The results show that gaze duration and total reading time were longer, and total number of fixations was greater in the divided-word presentation condition than in the line-final presentation condition. These results are consistent with Li et al. (2012) findings. More importantly, gaze durations and total reading time were also longer in the divided-word presentation condition than in the line-initial presentation condition. That fixation durations in the divided-word presentation condition were longer than in the other two presentation conditions strongly demonstrated the existence of the divided-word effect. This effect does not merely result from the differences in parafoveal processing. If less parafoveal processing induces longer fixation durations for the divided-word presentation condition than for the line-final presentation condition, it is anticipated that fixation durations are shorter for the divided-word presentation condition than for the line-initial presentation condition in which target words are presented in the peripheral vision without parafoveal processing. However, the results of the experiment are not consistent with this parafoveal processing explanation.

The results that divided words receive longer fixation durations are consistent with the prediction of the Chinese reading model (CRM; Li and Pollatsek, 2020). According to this model, character and word identifications interactively activate each other. The characters within the perceptual span are processed simultaneously. All words constituted by these characters are activated, and these spatial overlapping words compete for a winner. The activity in the word-processing levels feeds back to the character-processing level. So, character processing is not independent but is affected by word knowledge and the other character of the same word. When a two-character word is presented at the start or the end of a line, activation from both characters of this word can feed forward to the word-processing level, which makes word indentation more efficient. At the same time, the information from the word and the other character of the same word facilitates character recognition. However, when a two-character word is divided across two lines, the second character is in the peripheral vision, so word knowledge and the second character have less facilitation for the first character recognition. Another possible reason for the divided-word effect is that readers have to adopt a character-based strategy when a word is divided across lines, and store the character information in the working memory (Li et al., 2012, 2013). This extra working memory load may lead to longer fixation durations for the divided-word presentation than for the other presentation condition. What needs to be pointed out is that the CRM is constructed based on eye-movement data of reading a single-line sentence. At present, there are no eye-movement control models for multiline-line reading. To build CRMs for multiline reading, further studies about factors affecting the divided-word effect are needed.

The results that high-complexity words received longer fixation durations than low-complexity words are consistent with findings of studies in which target words are located at the intra-line position (Ma and Li, 2015; Li et al., 2019). According to multilevel interactive-activation architecture, word recognition begins with the extraction of characters’ stroke information (Taft et al., 1999), so it is no surprise that high-complexity words (as measured by the number of strokes) receive longer fixation durations.

As to the interaction between presentation condition and complexity, we found that the difference between the divided-word and the line-final presentation condition is marginally larger for low-complexity words than for high-complexity words on the total reading time. In the line-final presentation condition, visual simple words are more easily processed as a whole than visual complex words, but dividing a word across two lines disrupts this whole-word processing, so the divided-word effect is weaker for high-complexity words than for low-complexity words. It’s worth noting that the interaction between presentation condition and complexity only appears on the total reading time, but not on the gaze duration. Gaze duration reflects early word recognition processes, whereas total reading time reflects the integrative and inferential processes required for comprehension (Andrews and Veldre, 2019). So, it can be concluded that word complexity modulates the divided-word effect at the late stage of word recognition.

However, the differences between the divided-word presentation condition and line-initial presentation condition were similar for different complexity words. The reason may be that reader may not process line-initial words simultaneously as return-sweep landing positions are 5–8 letters away from the left text margin (Slattery and Vasilev, 2019; Vasilev et al., 2021). Thus, the processing advantage as a whole for low-complexity words compared to high-complexity words is smaller in the line-initial presentation condition than in the line-final presentation condition. More studies are needed to further explore this question in the future.

In summary, dividing a word across two lines interferes with reading, which has some implications for typesetting in Chinese. Dividing a word across two lines should be avoided as much as possible for improving reading efficiency. There are several ways to avoid splitting a word across two lines, such as left justified layout, and left and right justified layout. Considering Chinese characters are “square characters,” we should keep the text arranged neatly when keeping characters belonging to a word on the same line. So, the left and right justified layout may be better than the left justified layout.

## Data availability statement

The raw data supporting the conclusions of this article will be made available by the authors, without undue reservation.

## Ethics statement

The studies involving human participants were reviewed and approved by the research ethics committee in the Academy of Psychology and Behaviour at Tianjin Normal University. The patients/participants provided their written informed consent to participate in this study.

## Author contributions

MZ and XB designed the experiment. MZ collected the experiment data and conducted data analyses. All authors contributed to the article and approved the submitted version.

## Funding

This work was supported by the National Natural Science Foundation of China (no. 81471629) and Distinguished professor program of “Changjiang Scholar Award Program” of the Ministry of Education(no. T2017120) and The Postgraduate Innovative Research Projects of Tianjin (no. 2019YJSB132).

## Conflict of interest

The authors declare that the research was conducted in the absence of any commercial or financial relationships that could be construed as a potential conflict of interest.

## Publisher’s note

All claims expressed in this article are solely those of the authors and do not necessarily represent those of their affiliated organizations, or those of the publisher, the editors and the reviewers. Any product that may be evaluated in this article, or claim that may be made by its manufacturer, is not guaranteed or endorsed by the publisher.
